# Serum miR-497-5p serves as a diagnostic biomarker for acute coronary syndrome and predicts the occurrence of major adverse cardiovascular events after percutaneous coronary intervention

**DOI:** 10.1080/21655979.2022.2051885

**Published:** 2022-03-18

**Authors:** Tao Chen, Xueshan Zhang, Wei Qian, Ran Zhou, Mingyu Su, Yanfeng Ma

**Affiliations:** Department of Cardiology, Affiliated Hospital of Xuzhou Medical University, Xuzhou, China

**Keywords:** miR-497-5p, acute coronary syndrome, clinical biomarker

## Abstract

This study aimed to investigate the diagnostic value of microRNA (miR)-497-5p in acute coronary syndrome (ACS) and its predictive value for the occurrence of adverse major adverse cardiovascular events (MACEs). Real-time quantitative polymerase chain reaction (RT-qPCR) was performed to detect the expression of serum miR-497-5p in 110 ACS patients and 82 controls. And miR-497-5p levels were found to be significantly elevated in the patients (P < 0.001). Pearson correlation coefficient confirmed that miR-497-5p was positively correlated with Gensini scores (r = 0.684). The area under the Receiver-operating characteristic (ROC) curve was 0.861, which significantly identified patients with ACS, and was confirmed by logistic regression (OR = 8.533, 95%CI = 4.113–17.787, *P* < 0.001). Kaplan-Meier and Cox regression was performed to evaluate the predictive value of miR-497-5p in the occurrence of MACEs during a 6-month follow-up after percutaneous coronary intervention (PCI) in patients with ACS. The results demonstrated that miR-497-5p was an independent predictor of MACEs (HR = 4.773, 95%CI = 1.569–12.036, *P* = 0.013) and that patients with high level of miR-497-5p were more likely to develop MACEs after PCI (long-rank *P* = 0.019). Finally, miR-497-5p positively correlated with endothelial proinflammatory and adhesion factors. Our study suggests that serum miR-497-5p is a potential diagnostic marker for ACS and its elevated levels can predict a high risk of MACEs in ACS patients after PCI. And this may be associated with vascular endothelial injury.

## Graphical abstract

**Figure:**
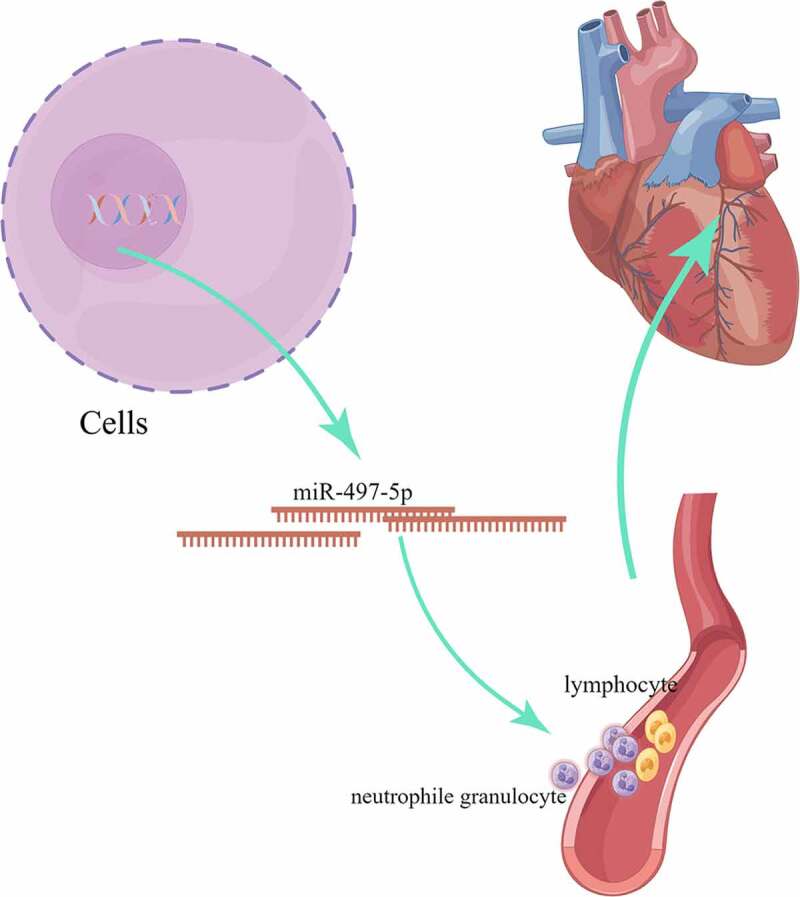

## Introduction

Acute coronary syndrome (ACS) is a clinical syndrome with rapid onset, progression, and high mortality rate [1]. It is mainly caused by myocardial ischemia with thrombus formation due to rupture of unstable atherosclerotic plaques or coronary arteries erosion [2]. The main clinical manifestations of ACS are acute myocardial infarction (AMI) and unstable angina pectoris (UA). Timely diagnosis and reperfusion therapy significantly reduce mortality in ACS [3]. Moreover, percutaneous coronary intervention (PCI) is an effective treatment to rapidly improve myocardial ischemia, restore perfusion, and reduce mortality in patients with ACS [4]. However, major adverse cardiovascular events (MACEs) such as angina pectoris, myocardial infarction (MI), heart failure, and death after PCI remain in 38% of ACS patients due to lack of effective monitoring [5]. Proactive search for timely and effective diagnostic biomarkers of ACS and prediction of MACEs after PCI is essential.

MicroRNAs (miRNAs) are non-coding RNA that can regulate cardiovascular disease by binding to target genes to inhibit the translational process. Furthermore, circulating miRNA has been discovered to be a useful biomarker for the diagnosis, prediction, and prognosis of cardiovascular disease due to its high conservation, sensitivity, and ease of detection. For example, miR-587 [6], miR-499 [7], and miR-204 [8] are promising biomarkers of ACS. MiR-497-5p is located on human chromosome 17p13.1 and has been reported to have a specific role in a variety of diseases. For example, elevated miR-497-5p is associated with heart failure progression [9]. Inhibition of miR-497-5p alleviated myocardial ischemia-induced dysfunction in mice [10]. Elevated serum miR-497 is a diagnostic and prognostic biomarker for atherosclerotic cerebral infarction [11]. Notably, upregulated miRNA in plaque arteries compared to normal coronary arteries contained miR-497 [12]. Furthermore, dysfunction of endothelial cell colony formation is associated with the development of coronary artery disease (CAD), and miR-497-5p was identified to be elevated in this cellular dysfunction [13]. However, ACS as the major pathogenic subtype of CAD, and the specific role of miR-497-5p in ACS is unclear.

Combining the evidence from this aforementioned research, we postulated that miR-497-5p is probably functioning in the ACS. Therefore, the focus of this study was to assess the diagnostic value of miR-497-5p in ACS and its predictive significance for the occurrence of MACEs within 6 months after PCI.

## Materials and methods

### Ethical statement

This present research was administered under the standards of the Declaration of Helsinki, and approaches were authorized by the Medical Council of Affiliated Hospital of Xuzhou Medical University (XYFY2018057). Informed consent was obtained from all subjects before the study.

### Study population

110 patients with ACS admitted to Affiliated Hospital of Xuzhou Medical University from June 2018 to January 2020 were selected. They were all diagnosed for the first time and coronary angiography showed at least one coronary artery stenosis requiring PCI. Among them included 72 patients with AMI and 38 patients with UA, aged 40–80 years. The diagnosis of AMI patients was based on the fourth edition of the Uniform Definition of AMI published by the European Society of Cardiology [14]. In addition, abnormal expression of cardiac markers such as cardiac troponin (cTnI)> 0.06 ng/ml was also confirmed AMI. UA patient’s diagnostic criteria were (1) clinical history; (2) mild exercise angina in the last few months; (3) chest pain lasting more than 20 min at rest; (4) no significant change in the markers of myocardial injury. Excluding criteria were: (1) malignant tumor or hematological disease; (2) congenital heart disease, or cardiogenic shock (3) those who have undergone cardiac surgery. 82 healthy subjects were matched to patients in the ACS group using propensity score matching among those who underwent health screening at our hospital during the same period. In addition, they had normal electrocardiogram, liver and kidney function, and individuals with tumors, autoimmune diseases, infectious diseases, and lactation were excluded.

### Data collection

Participants’ clinical baseline characteristics, including age, gender, body mass index (BMI), and common risk factors such as smoking, hypertension, hyperlipidemia, diabetes, as well myocardial injury marker cTnI, were recorded in Table 1. Hypertension was defined as measured > 140/90 mmHg [15]. Patients with a level of HbA1C >6.5%, fasting glucose >126 mg/dL, or using any oral anti-diabetic agents, insulin, or insulin analogs were defined as having diabetes. In addition, the severity of coronary atherosclerosis in patients with ACS was assessed using the Gensini score. Coronary angiography was performed by two experienced cardiologists via the femoral or radial artery, and each coronary lesion was scored for severity according to the importance and location of the stenosis to measure the patient’s coronary atherosclerosis burden [16]. Patients received loading doses of aspirin and antiplatelet agents including clopidogrel and ticagrelor before PCI. And 50–100 IU/kg of heparin was administered intravenously during PCI and received antiplatelet therapy after the procedure.

**Table 1.** General information of the enroll participants

Parameters	ACS patients (n = 110)	Controls (n = 82)	*P* values
Demographic			
Age, years	60.79 ± 9.01	61.82 ± 11.73	0.794
Gender, male, n (%)	60 (54.55)	42 (51.30)	0.664
BMI, kg/m^2^	24.19 ± 5.05	24.14 ± 2.40	0.943
Medical history			
Smoking, n (%)	60 (54.55)	34 (41.46)	0.081
Hypertension, n (%)	57 (51.82)	40 (48.78)	0.771
Diabetes mellitus, n (%)	49 (44.55)	39 (47.56)	0.770
Biomarkers			
TC, mmol/L	4.35 ± 0.56	4.20 ± 0.44	0.048
TG, mmol/L	1.62 (1.47, 1.77)	1.49 (1.30, 1.67)	0.001
HDL-C, mmol/L	0.94 (0.65, 1.23)	1.13 (0.92, 1.39)	0.000
LDL-C, mmol/L	2.65 (2.28, 3.00)	2.54 (2.02, 3.05)	0.248
Creatine, μmol/L	136.31 ± 12.12	135.26 ± 11.24	0.130
Hemoglobin, g/L	7.21 ± 0.65	5.27 ± 0.51	0.541
WBC, ×10^9^/L	7.12 ± 1.20	6.61 ± 0.98	0.002
BNP, pg/ml	434.51 ± 174.77	-	
cTnI, ng/mL	1.80 ± 0.50	-	
Gensini score	57.83 ± 15.09	-	

Note: BMI, body mass index; WBC, white blood cells, TC, total cholesterol; TG, triglyceride; HDL-C, high-density lipoprotein cholesterol; LDL, low-density lipoprotein cholesterol; FBG, fasting blood glucose; WBC, white blood cell; BNP, brain natriuretic peptide (BNP); cTnI, cardiac troponin I; Date was presented as mean ± SD, or median (first quartile, third quartile), or N (%).

### Serum sample collection

10 ml of venous blood from the upper limb of ACS patients were taken on admission (prior to PCI). At the same time, fasting blood was collected from the controls on the day of physical examination. After standing at room temperature, the supernatant was centrifuged at 1000 g for 15 min. The upper serum was stored at −80°C for further analysis.

### Follow-up program

The MACE occurring within 6 months after PCI in ACS patients was recorded by telephone, outpatient follow-up, or readmission. Cardiogenic death, unstable angina, revascularization, fresh heart failure, recurrent MI were used as follow-up event endpoints. Heart failure can be confirmed by clinical signs and symptoms examination, as well as cardiac ultrasound and chest X-ray. Patients with angina or MI can be diagnosed by ischemic symptoms, electrocardiogram, or elevated cardiac enzyme levels [17].

### Real-time quantitative polymerase chain reaction (RT-qPCR)

Serum samples were added to Trizol LS and total RNA was extracted. 500 ng RNA was synthesized by reverse transcription using the miRNA cDNA Synthesis Kit after analyzing its purity and quality. cDNA was synthesized by RT-qPCR using miRNA cDNA Assay Kit, template DNA, and primers. The reaction procedure was pre-denaturation at 95°C for 5 s, annealing at 60°C for 30s, extension at 72°C for 15s, and the reaction goes through 40 cycles. miRNA relative levels were measured using 2 ^−∆∆Ct^ and cel-miR-39 as a reference gene and normalized to miR-497-5p. Primers sequence used in the current study: miR-497-5p forward, 5’-ATCCAGTGCGTGTCGTG-3’ and reverse, 5’-TGCTCAGCAGCACACTGT-3’; cel-miR-39 forward, 5’-UCACCGGGUGAAAUCAGCUUG-3’ and reverse, 5’-TGCTCAGCAGCACACTGT-3’.

### Evaluation of inflammation and endothelial damage

Commercial enzyme-linked immunosorbent assay (ELISA) Kit was performed for measuring the concentrations of serum interleukin-1β (IL-1β), tumor necrosis factor (TNF-α), and endothelial damage markers intercellular adhesion molecule −1 (ICAM-1) and vascular cell adhesion molecule-1 (VCAM-1). Briefly, microtiter plates (96-well flat-bottom, with specific antibodies coated on the bottom) were coated for 24 h with samples diluted at a ratio of 1:2 in the diluent to a final volume of 100 μl. The samples were analyzed in duplicate. After incubation, the unbound antibody is washed off. Then the enzyme-catalyzed substrate was added and after incubation at room temperature, the termination solution was added. The OD value at 450 nm was measured and the concentration was calculated [18].

### Statistical analysis

SPSS 23.0 software was employed to statistically analyze the data and produce graphs. Shapiro-Wilk tests acquired the normality of the distribution of continuous variables, and when the normal distribution was satisfied, an independent student t-test was used to assess variability and performed as mean ± standard deviation (SD). Non-normally distributed data were expressed as medians (first quartile, third quartile) and statistical differences were analyzed using the Mann-Whitney U test. In addition, categorical variables were presented as frequency (percentage), and chi-squared tests were reviewed for analysis. ROC curves were assessed for the accuracy of miRNA in diagnosing ACS. Logistic regression was used to analyze the factors influencing the occurrence of ACS. Kaplan-Meier and Cox regression was employed to assess the predictive potential of miRNAs for MACEs. Pearson correlation coefficient was used to examine the relationship of miRNA and Gensini score or inflammatory factors or adhesion factors. *P* < 0.05 stated a statistically significant difference.

## Results

Previous studies reporting miR-497-5p dysregulation implied the potential clinical significance of miR-497-5p in ACS patients after PCI. To assess the clinical predictive value of miR-497-5p in ACS and to reveal its potential mechanisms, miR-497-5p was studied in patients’ serum.

### Baseline demographics

Clinical baseline characteristics of the subjects were presented in Table 1. 110 patients with ACS were enrolled, with a mean age of 60.79 ± 9.01 years and 54.55% males. The control group consisted of 82 cases with a mean age of 61.82 ± 11.73 years and 54.30% males. No statistical variation in smoking history, hypertension history, and diabetes history, as well as creatinine and low-density lipoprotein cholesterol (LDL-C) between the two groups (*P* > 0.05). However, patients with ACS had significantly higher total cholesterol (TC), triglyceride (TG), and white blood cell (WBC) than controls (*P* < 0.05), high-density lipoprotein cholesterol (HDL-C) was significantly lower than in controls (*P* < 0.001).

### Elevated miR-497-5p positively correlates with Gensini score in ACS patients

**Figure 1.**
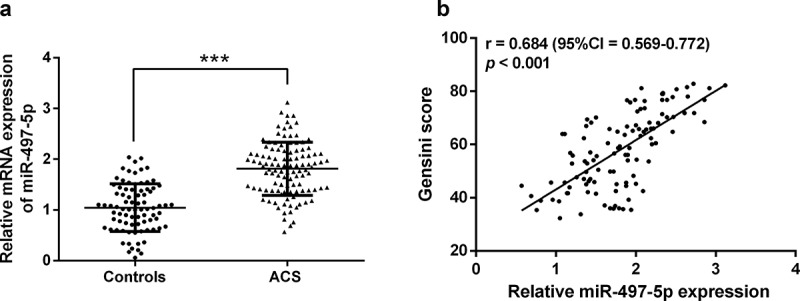
The serum expression of miR-497-5p in acute coronary syndromes patients and the correlation with Gensini score. (a) Real-time quantitative polymerase chain reaction (RT-qPCR) was performed to detect serum miR-497-5p levels in patients with acute coronary syndrome and healthy individuals. (b) In acute coronary syndromes patients, the Pearson correlation coefficient examined the relationship of miR-497-5p and Gensini score. ***P < 0.001 versus controls.

Serum miR-497-5p was first examined by RT-qPCR, which confirmed that miR-497-5p was significantly elevated in the serum of ACS patients than in controls (*P* < 0.05, Figure 1a). In addition, the Gensini scoring system provided a quantitative assessment of coronary artery stenosis severity [19]. Pearson’s correlation coefficient test exposed that serum miR-497-5p was positively correlated with Gensini score (r = 0.684, 95%CI = 0.569–0.772, Figure 1b).

### Serum miR-497-5p is a potential diagnostic biomarker for ACS

**Figure 2.**
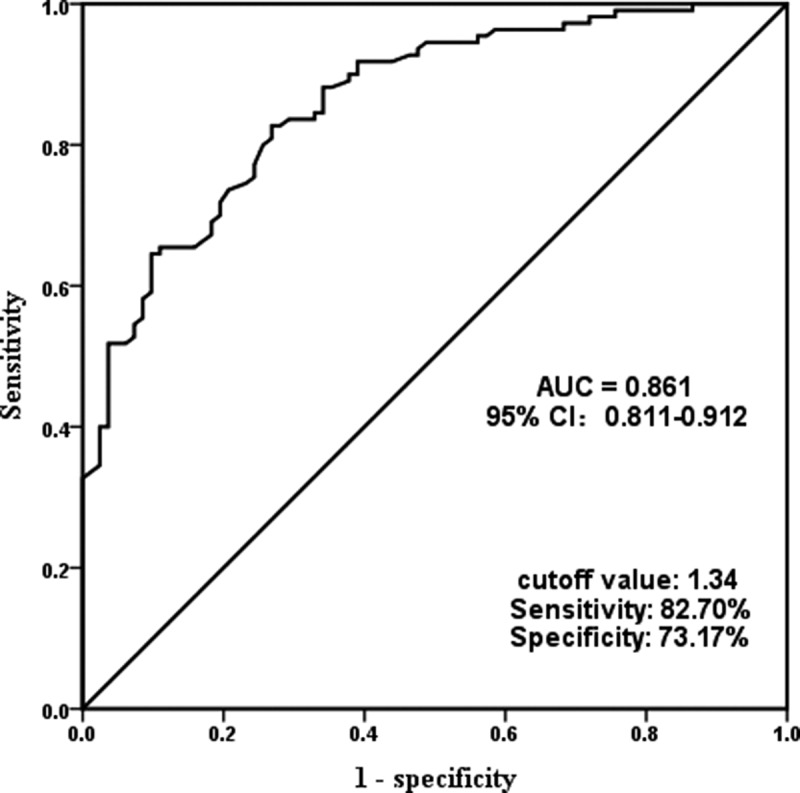
Receiver-operating characteristic (ROC) curves verified the clinical diagnostic value of miR-497-5p in predicting ACS.

To investigate the clinical importance of miR-497-5p, we plotted ROC curves based on serum miR-497-5p levels in the ACS group and the control group. As shown in Figure 2, serum miR-497-5p also distinguished well between ACS patients and controls at the cutoff value of 1.34, with an AUC of 0.861, a sensitivity of 82.70%, and a specificity of 73.17%. Subsequently, we included clinical parameters and miR-497-5p levels in ACS and controls in a logistic regression analysis of independent dichotomous variables to assess their independent effects on the development of ACS. Table 2 confirms that miR-497-5p can independently influence the incidence of ACS (OR = 8.553, 95% CI = 4.113–17.787).

**Table 2.** Association of different variables with the occurrence of ACS

Variables	OR	95% CI	*P* value
Age	0.944	0.459–1.942	0.875
Gender	0.862	0.425–1.748	0.680
BMI	0.871	0.425–1.784	0.705
Smoking	0.593	0.291–1.210	0.151
Hypertension	0.667	0.324–1.373	0.271
Diabetes mellitus	1.132	0.552–2.321	0.734
TC	0.720	0.352–1.472	0.368
TG	0.610	0.302–1.235	0.170
HDL-C	1.500	0.743–3.029	0.258
LDL-C	1.427	0.700–2.912	0.328
Creatine	0.767	0.373–1.576	0.470
Hemoglobin	1.181	0.584–2.390	0.643
WBC	1.831	0.896–3.742	0.097
**MiR-497-5p**	**8.553**	**4.113–17.787**	**0.000**

Note: BMI, body mass index; WBC, white blood cells, TC, total cholesterol; TG, triglyceride; HDL-C, high-density lipoprotein cholesterol; LDL, low-density lipoprotein cholesterol; FBG, fasting blood glucose; WBC, white blood cell.

### miR-497-5p predicts the occurrence of MACEs

**Figure 3.**
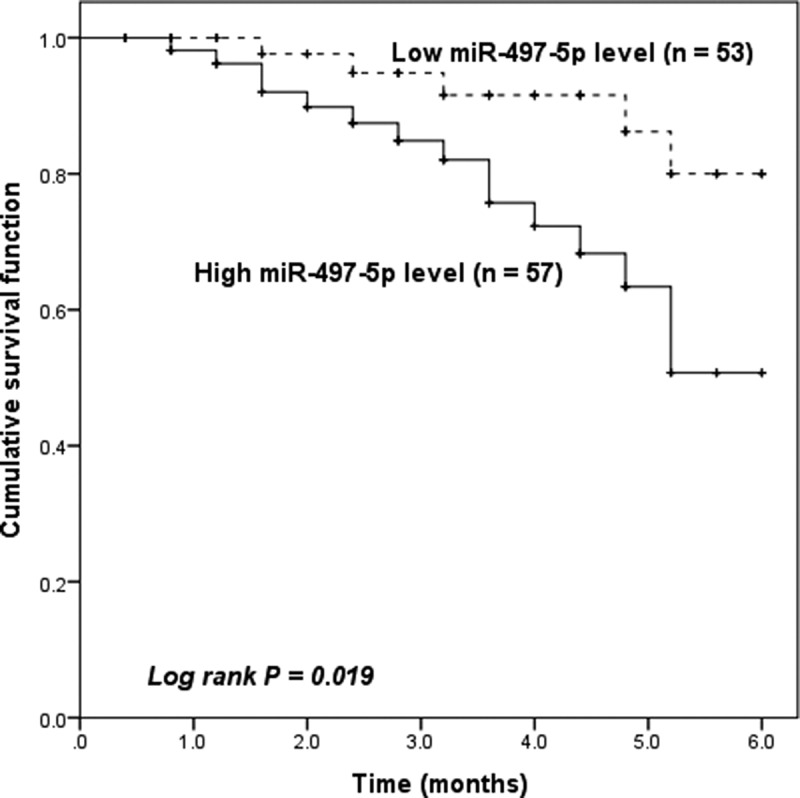
Kaplan-Meier survival curves based on serum miR-497-5p expression in ACS patients at 6 months.

Subsequently, we attempted to examine the predictive value of serum miR-497-5p for the occurrence of MACEs in patients after PCI. During the 6-month follow-up period, 20 patients (18.18%) developed MACEs, including 5 cases of unstable angina, 2 cases of heart failure, 9 cases of recurrent MI, and 4 cases of cardiac death. Finally, we grouped the ACS patients according to their mean miR-497-5p (1.81 ± 0.53) and plotted Kaplan-Meier curves. As seen in Figure 3, patients with high miR-497-5p expression were more experience MACEs after PCI (Log-rank, *P* = 0.019). Moreover, Cox regression test revealed that serum miR-497-5p was an independent predictor of the occurrence of MACEs in patients with ACS (HR = 4.773, 95% CI = 1.569–12.036, *P* = 0.013, Table 3).

**Table 3.** Multivariate Cox regression analysis of independent risk factors for MACE within 6 months in ACS patients

Characteristics	Multivariate analysis		
	HR	95%CI	*P*
**miR-497-5p**	**4.773**	**1.569–12.036**	**0.013**
Age	2.658	0.743–6.435	0.132
Gender	0.703	0.337–1.847	0.535
BMI	0.835	0.335–2.149	0.770
Smoking	1.374	0.658–3.895	0.563
Hypertension	1.898	0.243–1.537	0.253
Diabetes mellitus	0.581	0.289–1.614	0.340
TC	1.198	0.774–5.065	0.763
TG	0.862	0.627–4.338	0.825
HDL-C	0.899	0.308–1.841	0.858
LDL-C	0.497	0.296–1.734	0.279
Creatine	1.447	0.793–4.707	0.538
Hemoglobin	1.549	0.591–3.644	0.459
WBC	0.591	0.291–1.632	0.363
cTnI	4.664	1.222–9.099	0.028
NT-proBNP	3.052	1.107–8.225	0.068
Gensini score	3.405	0.885–5.780	0.041

Note BMI, body mass index; WBC, white blood cells, TC, total cholesterol; TG, triglyceride; HDL-C, high-density lipoprotein cholesterol; LDL, low-density lipoprotein cholesterol; FBG, fasting blood glucose; WBC, white blood cell; cTnI, cardiac troponin I; N-terminal pro-B-type natriuretic peptide, NT-proBNP.

### Elevated miR-497-5p correlates with vascular endothelial inflammation and adhesion factors

**Figure 4.**
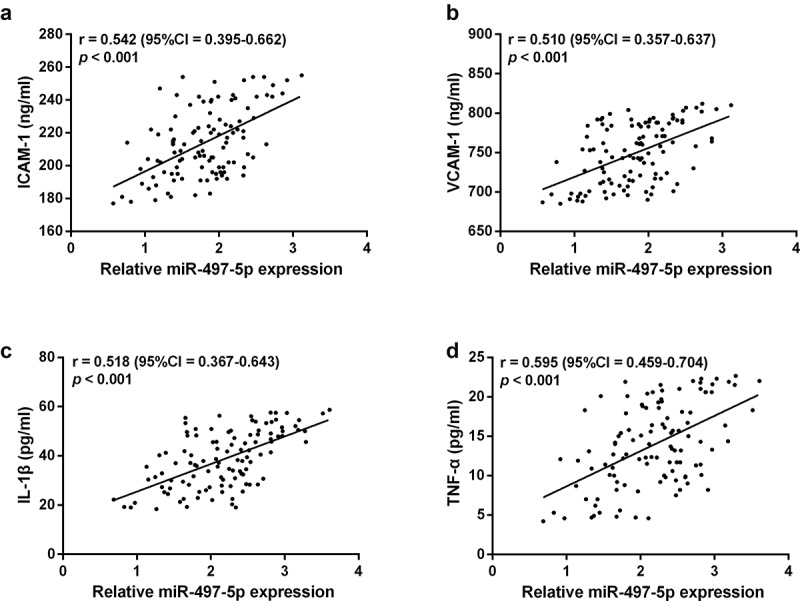
Correlation between serum miR-497-5p and endothelial adhesion factors ICAM-1 (a) VCAM-1 (b) as well as inflammatory factors IL-1β (c) and TNF-α (d) was evaluated by Person correlation coefficient.

Previous studies have confirmed that vascular endothelial inflammatory factors IL-1β, TNF-α, and adhesion factors ICAM-1 and VCAM-1 are essential markers of vascular endothelial injury and dysfunction, which can predict cardiovascular disease, and correlate with the severity of ACS [20]. It was confirmed that miR-497-5p showed a moderate positive correlation with ICAM-1 (r = 0.542), VCAM-1 (r = 0.510), IL-1β (r = 0.518) and TNF-α (r = 0.595, Figure 4), respectively.

## Discussion

In the current research, we discovered that serum miR-497-5p was generally elevated in patients with ACS and positively correlated with Gensini score as well as endothelial inflammatory factors and adhesion factors, respectively. Furthermore, elevated miR-497-5p is a potential diagnostic biomarker for ACS and can identify ACS patients from controls. Finally, patients with elevated miR-497-5p levels are more likely to develop MACEs after PCI and may serve as an independent predictor of MACE development.

ACS is a rapidly progressive cardiovascular disease with high mortality. The occurrence of ACS is currently assessed clinically through risk score algorithms, electrocardiography, angiography, myocardial enzyme [21]. However, they all have different drawbacks. For example, myocardial enzymes in patients with ACS are not diagnostic of unstable angina [22]. The currently used diagnostic marker cTnI has a low specificity [23]. Angiography is an invasive test with a risk of complications and does not easily screen a wide range of patients [24]. Therefore, patients with ACS need to find effective biological diagnostic and predictive markers to diagnose reversible myocardial ischemia before it progresses to necrosis so that clinicians can intervene on time.

There is now growing evidence that miRNAs can function as diagnostic, prognostic, and predictive biomarkers of ACS. For example, inhibition of miR-19b-1-5p is relative to the poor one-year MACE prognosis in patients with ACS [25]. Plasma miR-100 serves as a biomarker of plaque vulnerability in ACS [26]. miR-486-3p is a stable biomarker for ACS diagnosis [27]. miR-3646 distinguishes ACS patients from healthy volunteers and mediates the inflammatory response [28]. However, the usefulness of miR-497-5p in cardiovascular and cerebrovascular disease has been noted. miR-497-5p reduction attenuates myocardial dysfunction caused by ischemia and reperfusion [10]. Elevated serum miR-497-5p is associated with heart failure [9]. miR-497-5p elevation decreases inflammation and alleviated cerebral infarction in patients [29]. Abnormal expression of miR-497-5p in patients with atrial fibrillation [30]. What’s more, miR-497-5p expression is upregulated in coronary endothelial colony-forming cells [13]. Whereas the occurrence of diabetes increases the risk of ACS [31], and miR-497-5p is typically upregulated in diabetes patients [32]. What deserves our attention is ACS as a disease due to the rupture of coronary atherosclerotic plaque. Tian et al.’s research found that the expression profiles of differentially expressed miRNAs, including miR-497, in normal coronary arteries and arterial plaques were analyzed [13].

Based on the above analysis, we hypothesized that miR-497-5p may be functional in ACS patients. Therefore, the serum miR-497-5p levels of participants included in this study were significantly increased in ACS patients compared to controls. This is consistent with the expression levels of cardiovascular disease discussed above. What’s more, the Gensini score is a commonly used score to evaluate the degree of coronary stenosis and to quantify the severity of coronary atherosclerosis [33]. The higher the score, the more severe the proximal lesion in combination with the degree of stenosis and the site of stenosis [34]. In our research, it was noticed that elevated miR-497-5p in ACS patients was positively associated with increased Gencini score, indicating that the upregulation of miR-497-5p is correlated with the disease progression in ACS. We further analyzed the significance of miR-497-5p in the clinical diagnostic of ACS patients. ROC analysis demonstrated that miR-497-5p has high diagnostic potential and predictive ability as a potential biomarker of ACS, identifying ACS from controls. Moreover, logistic regression confirmed that miR-497-5p independently affected the occurrence of ACS.

PCI is effective in improving myocardial ischemia in patients with ACS, however, but is prone to MACEs post-procedure. Coronary angiography is expensive and time-consuming to monitor the occurrence of MACEs, therefore, finding other biomarkers to predict MACE is necessary. So, we followed up on the short-term occurrence of MACEs in patients after PCI and analyzed the predictive value of miR-497-5p. Kaplan-Meier analysis found that patients with higher miR-497-5p levels were more susceptible to MACE than those with lower miR-497-5p levels. Traditional cardiovascular risk factors such as age, history of heart disease, diabetes, hypertension, and dyslipidemia have all been shown to be associated with an increased incidence of MACEs and an increased risk of morbidity mortality and disability in patients with ACS [35]. In this study, Cox regression analysis identified miR-497-5p as an independent predictor of the occurrence of MACSs in ACS patients, but age, diabetes, and hypertension were not found to be significantly associated with MACEs, which may be due to the small sample size and the short follow-up period. In conclusion, our results confirm that miR-497-5p may serve as a novel noninvasive clinical predictor biomarker for ACS.

Finally, we tried to examine the specific mechanisms of miR-497-5p in ACS. The pathologic process of ACS is complex and includes the rupture of atherosclerotic plaques, platelet aggregation during thrombosis leading to complete or partial coronary artery occlusion, and a series of inflammatory reactions at plaque and thrombus sites leading to myocardial ischemia and necrosis [36]. Increased secretion of TNF-α and IL-1β in the inflammatory state activates the increase of endothelial adhesion factor. As cytokine-inducible members of the immunoglobulin gene superfamily, VCAM-1 and ICAM-1 are soluble endothelial adhesion factors, which are significantly upregulated in arterial inflammatory diseases. They promote monocyte adhesion and affect migration to endothelial cells, leading to plaque rupture and thrombosis [20]. In summary, they have been described as endothelial inflammation in the course of cardiovascular disease and associated with ACS severity [37]. However, in our study, we found that elevation of miR-497-5p was moderately positively associated with these endothelial inflammatory factors, that is, significantly positively associated with ACS severity. The results confirmed that miR-497-5p maybe participate in the progression of ACS by regulating endothelial inflammatory factors. There are some limitations to this research. Firstly, due to the limitations of the original data, patient recruitment failed to take into account the use of anti-hypertensive and anti-lipidemic medications. Secondly, further studies are needed to confirm whether miR-497-5p has been involved in the progression of ACS through endothelial inflammation. Furthermore, the present study provides the first evidence that circulating miR-497-5p can be served as a novel marker for ACS diagnosis and prediction of MACEs. Finally, the scope of this study was limited and more research is needed in a multicenter sample.

## Conclusion

Taking everything into account, our current study confirms that miR-497-5p is generally elevated in the serum of ACS patients. And miR-497-5p could be used as a novel diagnostic biomarker for ACS and predict the occurrence of MACEs after PCI.
